# Supplementary material for: Autophagy-Related 2 Regulates Chlorophyll Degradation under Abiotic Stress Conditions in Arabidopsis
Source: Int J Mol Sci. 2020 Jun 25;21(12):4515. doi: 10.3390/ijms21124515 (PMC7350272; doi:10.3390/ijms21124515)
Supplement: Supplementary file 1 [file ijms-21-04515-s001.pdf]

International Journal of
Molecular Sciences

MDPI

Supplementary Materials

Table S1. Genes Located in the 118.6 kb deletion region (*n* = 37).

Number	Locus	Location	Description
−1	AT3G19180	chloroplast	*The paralog of accumulation and replication of chloroplast 6* [1,2]
1	AT3G19184	nucleus	unknown
2	AT3G19190	nucleus	*Autophagy-related 2* [3]
3	AT3G19200	nucleus	unknown
4	AT3G19210	nucleus	*RAD54* [4]
5	AT3G19220	chloroplast	*Snowy cotyledon 2* [5]
6	AT3G19230	plasma membrane	unknown
7	AT3G19235	cellular component	unknown
8	AT3G19240	cytoplasm	unknown
9	AT3G19250	integral component of membrane	unknown
10	AT3G19260	Golgi apparatus	*Longevity assurance gene 1 homolog 2* [6]
11	AT3G19270	extracellular region	*Cytochrome P450* [7]
12	AT3G19274	mitochondrion	unknown
13	AT3G19280	Golgi apparatus	*Fucosyltransferase 11* [8]
14	AT3G03805	LncRNA	unknown
15	AT3G19290	nucleus	unknown
16	AT3G03815	LncRNA	unknown
17	AT3G19300	Plasma membrane	unknown
18	AT3G19310	plasma membrane	unknown
19	AT3G19320	non-hair root epidermal cell	unknown
20	AT3G19323	unknown	unknown
21	AT3G19330	nucleus	unknown
22	AT3G19340	endoplasmic reticulum	unknown
23	AT3G19350	cellular component	*Maternally expressed PAB C-terminal* [9]
24	AT3G19360	nucleus	unknown
25	AT3G19370	cytoplasm	unknown
26	AT3G19380	cytoplasm	*Plant U-box 25* [10]
27	AT3G19390	extracellular region	*Granulin repeat cysteine protease family protein* [11]
28	AT3G19400	cytosol	unknown
29	AT3G19410	cytoplasm	unknown
30	AT3G19420	cytosol	*Modified transport to the vacuole 3* [12]
31	AT3G19430	nucleus	unknown
32	AT3G19440	mitochondrion	unknown
33	AT3G19450	cytoplasm	*Cinnamyl alcohol dehydrogenase 4* [13]
34	AT3G19460	endoplasmic reticulum	unknown
35	AT3G19470	nucleus	unknown
36	AT3G19480	chloroplast	*Phosphoglycerate dehydrogenase 3* [14]

Table S2. Primers used in this study.

Primer Name	Sequence (5′–3′)
**Primers for mapping**	
M1-F	CAATGGGAAGAAGGTGTGAG
M1-R	CGCATTTCCATAAGTTTGTT
M2-F	ACCTGTTCAGTCTATGTTAC
M2-R	GGGAATTATTAACATTATCA
M3-F	ATGAGCTTTAGGAGTGTGTA
M3-R	AATTTTGTCCCAAAAGAATA
M4-F	TGACTGCTTGCACGTGAT
M4-R	CTCTCTTCCGCCGCGAAAT
M5-F	CTTTGCCATTATTTACTCTC
M5-R	CCAAAACCAAACTTAACTAT
M6-F	TGGAATTTACTCCTTATGAT
M6-R	GCAGAACCAATGAACATAC
M7-F	CGTCGTTTAGTGGATGTGTA
M7-R	CGGTGATAATGATTAATGAG
M8-F	CTTTTGTTCCTCATTTATGT
M8-R	TTCACGTTGTGGCGTTGTA
M9-F	GAATCTTGTAATCTGGTGC
M9-R	TACGTATACTTATTCAGTTGATC
M10-F	GTTCCATCCAAATCCTTAAT
M10-R	TTCCAAAATCAAAGTTATGT
M11-F	GTAGCCCAAAGCCGTACAG
M11-R	GAGATGCGTTTCACCTACAA
**Primers for the large fragment deletion test**	
F1	CTTTTGCTGGCTATGCTGCT
R1	AGCATCAGAAGGTGTTGATCT
R2	AGCAGCAAGTTCTCCTCTCA
**Primers for RT-PCR**	
AT3G19180-F	CTCAGAATTCCGTGGGCATG
AT3G19180-R	CTCTGTGGCCAATAACCTGC
AT3G19220-F	TTCCTCGTCTTCCTTCTCGC
AT3G19220-R	TCACTTCAATGTCCGTCCGA
AT3G19260-F	TCGATGGAGGTCTGGCATTT
AT3G19260-R	AGGGCTAGGATGATTGCTCC
AT3G19300-F	CTTCTACTGTGACCCTCCCC
AT3G19300-R	CGAGATGCAACGGATCACAG
AT3G19340-F	GGCTGACATGGAGATTGTGC
AT3G19340-R	AGTGTCCCGAGCAACTATCC
AT3G19380-F	ACAACCAGCTGATCCAACCT
AT3G19380-R	TTTTGAGAGCTCGTCGTGAC
AT3G19420-F	AGAATTGGCTGGGGACTTCA
AT3G19420-R	CCGGCTGTGATGATCCTTTG
AT3G19460-F	TCTTATGGGCCAAGTCTGCT
AT3G19460-R	TCTCTTTGTTTATGGGCAATGGA
AT3G19490-F	GGTGTGGTAGTGATAGCAGC
AT3G19490-R	AGTATCCCAGCTGCCTCAAG
Actin2-F	GGCTGGATTTGCAGGAGATG
Actin2-R	AAGGTCAAGACGGAGGATGG
**Primers for complementation**	
F	GGGGACAAGTTTGTACAAAAAAGCAGGCTTGATGGTGTTTCCGTGGAACA
R	GGGGACCACTTTGTACAAGAAAGCTGGGTTTATCGGTGTTGGTCCTGCT
**Primers for quantitative RT-PCR**	
Actin2-F	CCCGATGGGCAAGTCATC
Actin2-R	GAACAAGACTTCTGGGCATCTGA
CRD1(AT3G56940)-F	GGGTTAAACACAAAGGAGTTCGA

CRD1(AT3G56940)-F	GCCGTGGTTCGGTTTGTC
DVR(AT5G18660)-F	GTTTATAGCGGATTGCGTGTTG
DVR(AT5G18660)-R	TGGTCCACCAATTGGCAAA
CHLM(AT4G25080)-F	TCAAAAGCTACAAGGGCGTATCT
CHLM(AT4G25080)-R	ACTTTACCCAACGCTCTTTCCA
CHLH(AT5G13630)-F	GGGACACTTCCGCTGAAAAC
CHLH(AT5G13630)-R	TCTTGTCTTCCACCTGCGAGTA
CHLG(AT3G51820)-F	CCCTGTCAAATACGACGTCAAG
CHLG(AT3G51820)-R	TTCCGAGCACCAAGAATGG
PORA(AT5G54190)-F	GAGTCAGAGGCTGGGAAAAGAC
PORA(AT5G54190)-R	CCGACTTCGTCAAGCTTGGA
CAO(AT1G44446)-F	CGGATGTTAAACGGAGCAAAC
CAO(AT1G44446)-R	CCGAACTCCGAGCTTGTCAT
NYC1(AT4G13250)-F	CGAAGATTCAAGTGTACCCACAAG
NYC1(AT4G13250)-R	ACCCGTATCGGATCTCTGAAGA
NOL(AT5G04900)-F	GTCTGCGGCGGTGGAA
NOL(AT5G04900)-R	CGGAGGCGTCATAGGTTCTC
PPH(AT5G13800)-F	GAAAGCCGGTTGTGACAATCTC
PPH(AT5G13800)-R	CGCCAAAGCCAGGAAGAAA
ACD1(AT3G44880)-F	CACCGGCTCGCTCCTTTA
ACD1(AT3G44880)-R	CGAACATTGCAAGTGTCCATTC
ACD2(AT4G37000)-F	CTCATGTCGACGGTGGAGAA
ACD2(AT4G37000)-R	GAGGGAGGTTACAGGGAAGGA
NYE1(AT4G22920)-F	GGACCGGCGATTTTCGA
NYE1(AT4G22920)-R	TGCTTCTTCTCATCAACCCCTAA
HCAR(AT1G04620)-F	CAGTCAGTGCGGGCTATGC
HCAR(AT1G04620)-R	TGCACATGCCTCCTTCACA
TIR(AT1G57630)-F	CGCTTCTTCGTGGTGTTGTCT
TIR(AT1G57630)-R	CGTTTGACCCGACTCTTTCTTC
TIP1(AT2G43510)-F	TCCGATGTGCCGGAGATAG
TIP1(AT2G43510)-R	CACCTCCGTATTCTTTCAAGCA
SAP12(AT3G28210)-F	GCATTGCCAAGACCCTGATT
SAP12(AT3G28210)-R	GCAGCCGTCGCAAGTAAAAG
TPL(AT4G01870)-F	AACGGATGGGCCATGGA
TPL(AT4G01870)-R	GAATCCGATGAGATCTCCTTTCG
CV(AT2G25625)-F	GCGGGATTCAGATCAATGATG
CV(AT2G25625)-R	TTCTACCGGCGATTTCACAAC
ATG18A-F	GGCATGGATGGGAGCTTCTA
ATG18A-R	ACATTTCACCGCCGTTCAC
ATG5-F	TGAAGATGTACCGGAGATCGATAC
ATG5-R	GAACTCAACAGGGCGATTAAGG
ATG6-F	CACAGAGCTTCAACAAGCAAGAG
ATG6-R	CCCATTTGAGGTTGCAGAGAGT
ATG8A-F	GCCTCCAACTGCTGCATTG
ATG8A-R	GGAACCCATCCTCATCTTTGTG
ATG12-F	CAGGGACTGATAAATTTGCCAAA
ATG12-R	CATAGACAAACAACGAATCAGAATGA
**Primers for Yeast two-hybrid**	
ATG18A-F	GGGG ACA AGTTTGTACAAAAAAGCAGGCTCCATGGCCACCGTATCTTC
ATG18A-R	GGGGACCACTTTGTACAAGAA AGCTGG GTTTAGAAAACTGAAGGCGGTT
ATG18B-F	GGGG ACA AGTTTGTACAAAAAAGCAGGCTACATGGCGAATCTGTCCTC
ATG18B-R	GGGGACCACTTTGTACAAGAAAGCTGGGTTCACCCGGTGGTTATGG
ATG18C-F	GGGGACAAGTTTGTACAAAAAAGCAGGCTCAATGAGTTCAACTGTTTC
ATG18C-R	GGGGACCACTTTGTACAAGAAAGCTGGGTTCACGGGCGGTTGTCC
ATG18D-F	GGGGACAAGTTTGTACAAAAAAGCAGGCTCAATGGATCCTCGGAGA
ATG18D-R	GGGGACCACTTTGTACAAGAAAGCTGGGTCTATCTCGGGCTAT
ATG18E-F	GGGGACAAGTTTGTACAAAAAAGCAGGCTCAATGAATTCGATTGTCTCCA
ATG18E-R	GGGGACCACTTTGTACAAGAAAGCTGGGTTCACACCCCACCAACCATT

ATG18F-F	GGGGACAAGTTTGTACAAAAAAGCAGGCTTTATGAAGAAAAACGGTGA
ATG18F-R	GGGGACCACTTTGTACAAGAAAGCTGGGTCTAATCCACTTGCTCTTCA
ATG18G-F	GGGGACAAGTTTGTACAAAAAAGCAGGCTCGATGATGAAGAAGGGGAA
ATG18G-R	GGGGACCACTTTGTACAAGAAAGCTGGGTTTAATCACCTACAAAGGAA
ATG18H-F	GGGGACAAGTTTGTACAAAAAAGCAGGCTCTATGAAGAGTAACTCCAA
ATG18H-R	GGGGACCACTTTGTACAAGAAAGCTGGGTTCAACCTTCTTCGGAGAAA

Supplementary References

1. Glynn, J.M.; Yang, Y.; Vitha, S.; Schmitz, A.J.; Hemmes, M.; Miyagishima, S.Y.; Osteryoung, K.W. PARC6, a novel chloroplast division factor, influences FtsZ assembly and is required for recruitment of PDV1 during chloroplast division in *Arabidopsis*. *Plant J.* **2009**, *59*, 700–711.
2. Zhang, M.; Hu, Y.; Jia, J.; Li, D.; Zhang, R.; Gao, H.; He, Y. CDP1, a novel component of chloroplast division site positioning system in *Arabidopsis*. *Cell Res.* **2009**, *19*, 877–886.
3. Wang, Y.; Nishimura, M.T.; Zhao, T.; Tang, D. ATG2, an autophagy-related protein, negatively affects powdery mildew resistance and mildew-induced cell death in *Arabidopsis*. *Plant J.* **2011**, *68*, 74-87.
4. Hirakawa, T.; Hasegawa, J.; White, C.I.; Matsunaga, S. RAD54 forms DNA repair foci in response to DNA damage in living plant cells. *Plant J.* **2017**, *90*, 372-382.
5. Tanz, S.K.; Kilian, J.; Johnsson, C.; Apel, K.; Small, I.; Harter, K.; Wanke, D.; Pogson, B.; Albrecht, V. The SCO2 protein disulphide isomerase is required for thylakoid biogenesis and interacts with LHCB1 chlorophyll a/b binding proteins which affects chlorophyll biosynthesis in *Arabidopsis* seedlings. *Plant J.* **2012**, *69*, 743–754.
6. Luttgeharm, K.D.; Chen, M.; Mehra, A.; Cahoon, R.E.; Markham, J.E.; Cahoon, E.B. Overexpression of *Arabidopsis* Ceramide Synthases Differentially Affects Growth, Sphingolipid Metabolism, Programmed Cell Death, and Mycotoxin Resistance. *Plant Physiol.* **2015**, *169*, 1108–1117.
7. Liu, R.; Liu, Y.; Ye, N.; Zhu, G.; Chen, M.; Jia, L.; Xia, Y.; Shi, L.; Jia, W.; Zhang, J. AtDsPTP1 acts as a negative regulator in osmotic stress signalling during *Arabidopsis* seed germination and seedling establishment. *J. Exp. Bot.* **2015**, *66*, 1339–1353.
8. Both, P.; Sobczak, L.; Breton, C.; Hann, S.; Nobauer, K.; Paschinger, K.; Kozmon, S.; Mucha, J.; Wilson, I.B. Distantly related plant and nematode core alpha1,3-fucosyltransferases display similar trends in structure-function relationships. *Glycobiology* **2011**, *21*, 1401–1415.
9. Qiu, Y.; Liu, S.L.; Adams, K.L. Frequent changes in expression profile and accelerated sequence evolution of duplicated imprinted genes in *Arabidopsis*. *Genome Biol. Evol.* **2014**, *6*, 1830–1842.
10. Wang, J.; Grubb, L.E.; Wang, J.; Liang, X.; Li, L.; Gao, C.; Ma, M.; Feng, F.; Li, M.; Li, L.; et al. A Regulatory Module Controlling Homeostasis of a Plant Immune Kinase. *Mol. Cell* **2018**, *69*, 493–504 e6.
11. Rustgi, S.; Boex-Fontvieille, E.; Reinbothe, C.; von Wettstein, D.; Reinbothe, S. Serpin1 and WSCP differentially regulate the activity of the cysteine protease RD21 during plant development in *Arabidopsis thaliana*. *Proc. Natl. Acad. Sci. USA* **2016**, *114*, 2212–2217.
12. Pribat, A.; Sormani, R.; Rousseau-Gueutin, M.; Julkowska, M.M.; Testerink, C.; Joubes, J.; Castroviejo, M.; Laguerre, M.; Meyer, C.; Germain, V.; et al. A novel class of PTEN protein in *Arabidopsis* displays unusual phosphoinositide phosphatase activity and efficiently binds phosphatidic acid. *Biochem. J.* **2012**, *441*, 161–171.
13. Aborode, F.A.; Raab, A.; Voigt, M.; Costa, L.M.; Krupp, E.M.; Feldmann, J. The importance of glutathione and phytochelatins on the selenite and arsenate detoxification in *Arabidopsis thaliana*. *J. Environ. Sci.* **2016**, *49*, 150–161.
14. Benstein, R.M.; Ludewig, K.; Wulfert, S.; Wittek, S.; Gigolashvili, T.; Frerigmann, H.; Gierth, M.; Flugge, U.I.; Krueger, S. *Arabidopsis* phosphoglycerate dehydrogenase1 of the phosphoserine pathway is essential for development and required for ammonium assimilation and tryptophan biosynthesis. *Plant Cell* **2013**, *25*, 5011–5029.

.
